# Caregiver and healthcare professional perspectives on drivers of routine immunisation uptake in East New Britain, Papua New Guinea: a qualitative study

**DOI:** 10.1136/bmjph-2025-003553

**Published:** 2026-03-18

**Authors:** Milena Dalton, William Pomat, Benjamin Sanderson, Pele Melepia, Hannah A James, Delmah Burangat, Lynette Duwaba, Daniel Willie, Benjamin Paivu, Patrick Kiromat, Elsie Stanley, Edward Waramin, Moses Laman, Shelley Walker, Caroline Homer, Leanne J Robinson, Michelle J L Scoullar, Stefanie Vaccher, Margie Danchin

**Affiliations:** 1 Burnet Institute, Melbourne, Victoria, Australia; 2 The University of Melbourne Faculty of Medicine Dentistry and Health Sciences, Melbourne, Victoria, Australia; 3 Papua New Guinea Institute of Medical Research, Goroka, Papua New Guinea; 4 University of New South Wales, The Kirby Institute, Sydney, New South Wales, Australia; 5 Mustard Seed Global, Glenmore Park, New South Wales, Australia; 6 Burnet Institute, Kokopo, Papua New Guinea; 7 East New Britain Provincial Health Authority, Kokopo, Papua New Guinea; 8 Government of Papua New Guinea National Department of Health, Port Moresby, Papua New Guinea; 9 Papua New Guinea Institute of Medical Research, Port Moresby, Papua New Guinea; 10 National Drug Research Institute, Curtin University, Perth, Western Australia, Australia; 11 The School of Public Health and Preventive Medicine, Monash University, Melbourne, Victoria, Australia; 12 Vaccine Uptake Group, Murdoch Childrens Research Institute, Parkville, Victoria, Australia; 13 Department of General Medicine, Royal Children’s Hospital Melbourne, Parkville, Victoria, Australia

**Keywords:** Vaccination, Community Health, Health Personnel, Public Health, Qualitative Research

## Abstract

**Introduction:**

Immunisation coverage varies across Papua New Guinea (PNG), with 40% of children in East New Britain Province (ENBP) classified as zero-dose (children missing diphtheria-tetanus-pertussis vaccine first dose (DTP1)) and 55% as under-immunised (missing DTP3) in 2023. This study aimed to understand routine childhood immunisation enablers and barriers in areas of ENBP reported to have highly variable DTP1, DTP3 and measles-rubella first-dose (MR1) coverage.

**Methods:**

Qualitative in-depth semi-structured interviews were conducted face-to-face with caregivers of children aged 9–23 months and healthcare professionals in Gazelle and Kokopo districts. Local research officers conducted interviews in Tok Pisin and English. Data were thematically analysed and mapped across the six UNICEF Journey to Health and Immunisation framework stages.

**Results:**

33 caregivers and 28 healthcare professionals were interviewed. Despite finding that a high proportion of caregivers’ children had received DTP, DTP3 and MR1, participants identified a myriad of contextual caregiver and healthcare professional enablers and barriers to routine immunisation. Caregiver enablers included support provided by families and community-based services; barriers included limited immunisation knowledge, financial and travel challenges, and limited availability of healthcare professionals. Healthcare professional enablers included increased operational funds and working with community leaders, while barriers were focused on service delivery including costs, non-functioning aid posts and vaccine stockouts. Strategies suggested by participants for improving vaccine uptake included developing tailored immunisation communication materials, engaging community members to support routine immunisation promotion activities, and strengthening coordination between communities and healthcare professionals.

**Conclusions:**

This study highlights the challenge of identifying and including zero-dose children and their caregivers in research but still provides useful contextual focus points to enhance and inform tailored strategies to strengthen immunisation service delivery. These include strategies that improve bidirectional coordination and communication between healthcare professionals, community and religious leaders about routine immunisation, increase immunisation knowledge and strengthen community-based services for vaccine delivery.

**WHAT IS ALREADY KNOWN ON THIS TOPIC**

- Reaching zero-dose children (children missing first-dose diphtheria-tetanus-pertussis (DTP1)) and under-immunised children (missing DTP3) is the cornerstone of the 2030 Immunisation Agenda which provides a strategic framework to maximise the impact of life-saving vaccines.
- In 2023, 44% of children were considered zero-dose and 59% were under-immunised in Papua New Guinea (PNG). In East New Britain Province (ENBP), PNG, 40% of children were estimated to be zero-dose and 55% under-immunised in 2023.
- A cross-sectional assessment of front-line services conducted in 2016–2017 in ENBP identified key immunisation programme performance strengths and weaknesses. Areas to strengthen included understanding of the population catchments, reach and efficiency of outreach services, staff knowledge of vaccination and community engagement.

**WHAT THIS STUDY ADDS**

- This study provides new insights into contextual enablers and barriers to routine childhood immunisation access and uptake in ENBP, and strategies that can be employed to strengthen enablers and address barriers to immunisation coverage. It also highlights the challenge in identifying and including caregivers of zero-dose children, and the strong interest community members have in increasing their immunisation knowledge.

**HOW THIS STUDY MIGHT AFFECT RESEARCH, PRACTICE OR POLICY**

- This study emphasises the importance of prioritising time for clear communication and discussion during the provider-recipient interactions to respond to caregivers’ questions around routine immunisation, demonstrates the value in using research to work with communities and identify focus areas to adjust approaches, and directly informs strategies to strengthen routine childhood immunisation practice.
- Findings from this study have already informed activities to increase routine childhood immunisation coverage. This includes developing context-specific information, communication and education materials as well as training programmes that meet the specific needs of the community and healthcare professionals in ENBP.

## Background

 Vaccines are one of the most effective disease prevention interventions, saving at least 154 million lives over the past 50 years.^1 2^ Service interruptions from the COVID-19 pandemic (2020–2023) caused a 5% global drop in childhood immunisation coverage for third-dose diphtheria-tetanus-pertussis vaccine (DTP3), from 86% in 2019 to 81% in 2021.^2^ Recent efforts to regain lost ground increased global DTP3 coverage to 84% in 2023.^3^ However, large numbers of children remain at risk, with 14.5 million children globally not receiving the DTP1 in 2023 (herein referred to as zero-dose children), and a further 6.5 million who are under-immunised children (those who had not received DTP3 necessary for full protection).^3^

In Papua New Guinea (PNG), 44% of children were estimated to be zero-dose in 2023, 59% under-immunised and 46% were missing their first-dose measles-rubella vaccine (MR1).^4 5^ East New Britain Province (ENBP), an island region located in the northeast of PNG, had substantial increases in immunisation coverage between 2019 and 2020 for DTP3 which increased from 53% to 65% and MR1 which increased from 36% to 64%.^5 6^ However, DTP1 declined from 67% in 2019 to 60% in 2020, and DTP3 and MR1 coverage rates have declined in subsequent years. In 2023 DTP3 and MR1 coverage rates were estimated to be 45% leaving a greater number of children susceptible to vaccine preventable diseases.^5 7^ These rates are substantially below the WHO PNG target of 80%.^5^ Families in PNG face complex barriers to accessing routine vaccines, including health system resource constraints, geographical isolation, socioeconomic challenges and socio-cultural factors, many of which were exacerbated by the COVID-19 pandemic.^8 9^ Geographical accessibility remains one of the biggest barriers, with 87% of the PNG population living in remote areas.^10^

Research and innovation are needed to inform evidence-based strategies to support vaccine acceptance, access to immunisation services, health service delivery and the vaccine supply chain to improve uptake.^11^ After identifying the target areas, identifying context-specific enablers and barriers in these areas is the next step in developing tailored strategies to improve vaccination coverage.^12 13^ The aim of this paper is to report on qualitative findings of a mixed methods study exploring enablers, barriers and strategies to reduce these barriers to routine childhood immunisation in ENBP.^14^

## Research methods

### Study design

Between February and September 2023, we conducted in-depth semi-structured interviews with caregivers of children aged 9 to 23 months and healthcare professionals from nine health facilities in two districts of ENBP reported to have highly variable DTP1, DTP3 and MR1 immunisation coverage.^8^ We also interviewed ENB Provincial Health Authority (PHA) management involved in immunisation service provision. To inform project design and contextual relevance, key stakeholders were consulted throughout the project, including ENB PHA management, community and religious leaders, government officials, academics with public health and immunisation expertise, and paediatricians from both PNG and Australia.

### Study setting

ENBP is the most populous province in the Islands Region of PNG with a population of approximately 457 169, with 65% of the population in Kokopo and Gazelle districts where this study was conducted.^15^ Five local-level government (LLG) areas were purposively selected for inclusion based on health facility coverage, population growth estimates and stakeholder consultations. Included LLGs were Inland Baining Rural and Toma-Vunadidir Rural (Gazelle District), and Bitapaka Rural, Duke of York Rural and Kokopo/Vunamami Urban (Kokopo District), with some locations within these districts excluded due to law-and-order concerns. In ENBP, health facilities aim to conduct one facility-based immunisation clinic per week and are encouraged to provide immunisation services every day, quarterly one-day mobile outreach clinics and overnight outreach patrols in the community.^9 16 17^

### Participants

Participant eligibility for caregivers included being 18 years or older, a primary caregiver of a child aged 9 to 23 months and residing in a study area. If a caregiver had multiple children aged between 9 and 23 months, they only reported on their oldest child within that age range.^18^ To minimise selection bias, caregivers were randomly recruited from selected villages for the survey component of the cross-sectional study by visiting every second house and asking eligible caregivers if they were interested in participating.^18^ To do this, the research team met with a community leader to facilitate entry into the community. We aimed to conduct at least five interviews per LLG area with both caregivers of immunised children and caregivers of under-immunised children, to increase the potential for a broad range of views and experiences. However, logistic, security and resource challenges limited this approach, and we suspected that those with unvaccinated children would be less likely to participate. Interviews were conducted at caregivers’ homes or in areas chosen to facilitate privacy within community meeting places. One caregiver who could not read or write was excluded because an appropriate witness was not available to witness verbal consent.

Healthcare professionals were eligible if they provided immunisation services or were an Officer in Charge (senior health facility management) at one of the nine study area health facilities or involved in ENB PHA immunisation service provision management. We aimed to conduct at least three interviews per health facility and two with ENB PHA management. Interviews were conducted in private spaces with facility-based healthcare professionals at the relevant health facility and interviews with PHA management were conducted at the PHA. Interviews with caregivers and healthcare professionals lasted about 30 minutes.

### Data collection

Two semi-structured interview guides (online supplemental file 1), one for caregivers and another for healthcare professionals, were developed in English, translated into Tok Pisin by a PNG translation company, and further adapted to the local context in consultation with local researchers, healthcare professionals and community members. Interview guides were informed by previous guides used to capture social and behavioural insights to routine childhood immunisation, including items from the WHO Behavioural and Social Drivers of vaccination tool.^19^,^22^ Qualitative interviews were conducted face-to-face by five Papua New Guinean research officers, HAJ, LD, DB, DW and BP (three female and two male) who received qualitative research and ethics training from PM, MiD, BS, SW, MaD across two workshops. The second workshop included informed consent and interview role play and a discussion to reflect on beliefs or expectations that may influence the interview to reduce interviewer bias and support inter-rater reliability.

Interviews were conducted in Tok Pisin, Kuanua or English depending on the participant’s preference and audio recorded. Transcripts were not returned to participants for review due to logistical constraints.

### Public involvement

Prior to caregiver data collection, research officers informed communities within the study areas during community or religious gatherings about the study and discussed the opportunity for them to participate. For healthcare professionals, a letter from the PHA CEO outlining the study was delivered to each of the health facilities and a member of the research team discussed the study and opportunity for health facility staff to participate with the Officer in Charge prior to data collection. For PHA management, the research team discussed the study and opportunity for participation. We piloted interview guides in one rural and one urban study area with two caregivers and two healthcare professionals. Using observations from the pilot testing, further context-specific adaptations were made to the interview guides in consultation with healthcare professionals, community members and local researchers.

### Data analysis

Tok Pisin language audio recordings were transcribed verbatim by a PNG translation company and then translated into English for analysis. English language recordings were transcribed using an online transcription service. Tok Pisin and English transcripts were cross-checked by a member of the research team. Data were analysed thematically. NVivo qualitative data software was used to organise and manage the transcripts. MiD and BS developed deductive codes based on interview topics and relevant literature on enablers and barriers to routine childhood immunisation. For consistency and rigour, two members of the research team (MiD and BS) independently coded three transcripts and any discrepancies were discussed, leading to the finalisation of overarching themes and sub-themes, which were discussed with the local research team. MiD and BS then applied these to the remaining transcripts.^23^

A series of consultative meetings and workshops were held with the research officer team, investigator team, ENB PHA senior management and Project Advisory Panel, to discuss and validate the analysis and findings, sense checking for interpretation and relevance. All five research officers who conducted the interviews were Papua New Guinean, with one officer, DB from ENBP. Four of the research officers were healthcare professionals. The investigator team which oversaw the study design and implementation comprised senior ENB PHA senior management, a paediatrician from ENBP, two Australian paediatricians and Papua New Guinean and Australian social science health and immunisation researchers providing a mix of local and international subject matter expertise. Engagement with senior management from the ENB PHA ensured that the study design, implementation and interpretation of results were relevant to the ENBP context. The Project Advisory Panel comprised PNG child health and immunisation researchers, PNG and ENBP immunisation programme managers, as well as Australian immunisation and child health researchers with extensive experience conducting research in the province, providing diverse insights during a discussion of key findings.

Identifiable information has been removed from participant quotes. Each quote is labelled with the participant’s category (caregiver or healthcare professional) and geographical location (urban or rural). We have used the consolidated criteria for reporting qualitative (online supplemental file 4) research to present our findings.^24^

### Reflexivity statement

The way in which the interviews were conducted, including probing, is influenced by the positionality of the five interviewers described above. The MiD who led the analysis was a principal investigator in this study as part of her immunisation and health systems strengthening PhD research. MiD and BS, both Australian immunisation and health systems strengthening researchers, acknowledge that the data analysis is influenced by this positionality. MiD lived and worked in PNG prior to commencing this study, and both MiD and BS travelled to ENBP several times during the study. This enabled some contextual reflexivity. In recognition of this, the analysis and interpretation of findings was discussed and informed by the PNG research officer team and key stakeholders as described above. This partnership approach acknowledged that expertise from multiple backgrounds and perspectives can inform solutions to complex problems (online supplemental file 3).^25^

### UNICEF journey to health and immunisation framework

Once data was thematically analysed, findings were applied to the UNICEF Journey to Health and Immunisation framework (online supplemental file 2).^26^ This framework is a conceptual model for understanding factors influencing caregivers’ and healthcare professionals’ decisions and actions around childhood immunisation, including identifying enablers and barriers. It maps out the journey in six stages and provides a guide to understanding the decision-making process and behaviours of caregivers and healthcare professionals within a broader ecosystem during the entire immunisation process from awareness to post-vaccination follow-up.^26 27^

## Results

Participant characteristics are outlined below. Qualitative data is presented via the six stages of the UNICEF Journey to Health and Immunisation framework (including enablers and barriers to routine childhood immunisation) followed by strategies for enhancing enablers and addressing barriers.

### Participant characteristics

Thirty-three caregivers and 28 healthcare professionals participated in interviews, reaching thematic data saturation (table 1). Most caregivers lived in rural areas, where the nearest health facility was between five and 80 minutes’ walk; five were male and 28 were female. All 33 caregivers reported that their child had received DTP1, 30 reported their child had received DTP3 and 32 reported their child received MR1 (30 through record book check and three through verbal recall). Around a third of healthcare professionals worked in an urban area and the remainder in rural areas; five were male and 23 were female.

**Table 1:** Participant characteristics

Participant group	Caregiver (%) (n=33)	Healthcare professionals (%) (n=28)
Local level government area		
Kokopo/Vunamami Urban	5 (15.1)	9 (32.1)
Bitapaka	13 (39.4)	2 (7.1)
Duke of York	9 (27.3)	6 (21.4)
Toma-Vunadidir	3 (9.1)	5 (18)
Inland Baining	3 (9.1)	6 (21.4)
Sex		
Male	5 (15.2)	5 (17.9)
Female	28 (84.8)	23 (82.1)
Reported child vaccination coverage		
DTP1	33 (100)	N/A
DTP3	30 (91)	N/A
MR1	32 (97)	N/A

DTP, Diphtheria tetanus toxoid and pertussis; MR1, measles-rubella first-dose.

#### Enablers and barriers from the perspective of caregivers and healthcare professionals

##### Stage 1: knowledge, awareness and beliefs

Almost all caregivers voiced their support for immunisation and understood that vaccines protect children from vaccine preventable diseases.

I believe in child vaccination because it is the only way that helps my children avoid illness. I am always up to date with my child’s immunisation to protect them from illnesses. (caregiver Kokopo/Vunamami Urban - urban)

**Figure 1:**
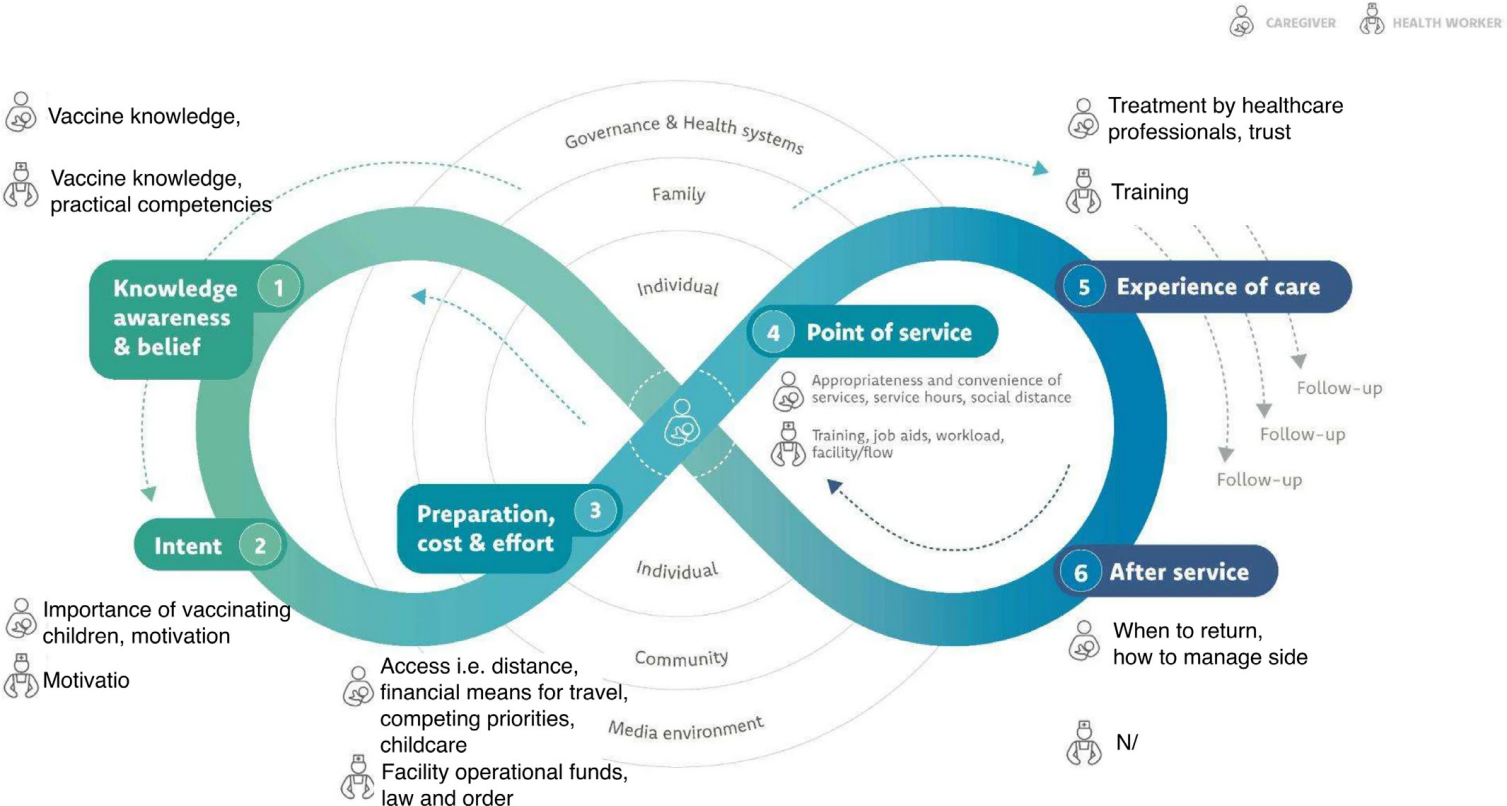
Participating caregivers and healthcare professionals’ journey to immunisation, adapted from the UNICEF Journey to Health and Immunisation framework.^26^

However, when asked to discuss reasons why some caregivers do not vaccinate their child(ren), some healthcare professionals perceived caregivers as unknowledgeable about vaccinating their children and believed they had limited support from husbands (tables2  3 and figure 1). One urban-based healthcare professional posited that *“maybe they were not given the health importance or health education about the importance of the vaccination”.* One rural-based caregiver from Duke of York islands echoed this by saying that *“baby’s vaccination is good but sometimes we are scared of the other diseases that they talk about, so it stops us from going to get routine immunisation, that is all”.* COVID-19 vaccine myths and misinformation were reported to have led to less caregivers bringing their child for routine childhood vaccines. Caregivers reported that understanding routine immunisation information, education and communication materials can be challenging for those with lower levels of education.

**Table 2:** Key immunisation enablers and barriers for caregivers, mapped to the UNICEF Journey to Health and Immunisation framework^26^

Knowledge, awareness and belief	Intent	Preparation, cost and effort	Point of service	Experience of care	After service
Enablers					
Understanding vaccines protect children	Motivated by wanting a healthy child	Family and community support	Health education sessions Door-to-door approach	Caregivers satisfied with village-based services	Recording the next vaccination date in the child health record book
Barriers					
*Caregivers do not understand the importance and effectiveness of childhood vaccination for preventable diseases *Husbands are unsupportive of mothers vaccinating child(ren) Mothers find educational immunisation materials (ie, posters and leaflets) hard to understand due to low literacy levels Myths and misinformation about the COVID-19 vaccine meant some caregivers did not want their child to receive routine vaccines	Poor understanding of childhood vaccination importance means some caregivers are unmotivated to vaccinate child(ren)	Transport to health facilities is often unaffordable Health facilities are sometimes too far away to travel Concerns about vaccine availability at some health facilities Weather events, road and sea conditions make it hard to travel Women with multiple children find it hard to find childcare and cannot carry multiple children Familial support influences available finances to access health services Caregivers are unable to take time off work Single mothers lack support to access vaccination services Women do not feel safe travelling to health facilities with law-and-order issues in the community *Population movement from intermarriages and plantation work leads to delayed vaccination	Not enough clinic staff to give vaccines or conduct outreach Vaccine supply stockouts Power outages Outreach services not well-publicised (eg, communities are not aware that the healthcare professionals are coming to their community before they arrive) Healthcare professionals do not take the time to explain why children should have the vaccines at the time of vaccination	Caregivers feel judged by healthcare professionals (eg, if they do not have the child’s health record book with them, are not able to answer questions or are a mother who gave birth in their village for fear they will get told off for not having delivered at a health facility) Vaccine stockouts lead to distrust of healthcare professionals	Healthcare professionals do not write the next vaccination date in the caregiver’s child health record book *Do not know how to manage vaccine side effects as insufficient information on vaccine side effects is provided

* From the perspective of healthcare professionals.

**Table 3:** Key enablers and barriers for healthcare professionals providing immunisation services, mapped to the UNICEF Journey to Health and Immunisation framework^26^

Knowledge, awareness and belief	Intent	Preparation, cost and effort	Point of service	Experience of care	After service
Enablers					
N/A	N/A	Increased operational funding during immunisation campaigns A vehicle for outreach Liaising with community leaders ahead of outreach and mobile patrols	Door-to-door approach	Immunisation training	N/A
Barriers					
View caregivers as unknowledgeable about vaccinating their child(ren) *Inadequate explanation of the purpose of vaccines or practical competencies to vaccinate	Inadequate human resources, vaccine stock and vaccine equipment to provide quality immunisation services impacts healthcare professional motivation for service delivery	Financial capacity of the health facility to provide routine immunisation services including outreach and mobile patrols (ie, no vehicle, boat or fuel available for healthcare professionals to use) Charging 1 Kina for vaccination services Law-and-order issues prevent healthcare professionals from reaching some communities as they feel unsafe	Non-maternal and child healthcare professionals are not confident to carry out immunisation activities if maternal and child healthcare professionals are not present Mothers forget to bring the child health book with vaccination records which sometimes leads to the child not receiving additional vaccines Inadequate facility infrastructure to provide quality immunisation services Caregivers are not in their villages when healthcare professionals conduct outreach *Health education sessions are not being run in some areas Vaccine stockouts	Healthcare professionals need refresher immunisation training Some healthcare professionals do not fully explain what vaccines are for or what diseases they prevent	N/A

* From the perspective of the caregiver.

Most mothers here are like me, I am one of those who just completed grade 1 and some mothers here do not know how to read and write, and we just live like that. We do not know about routine immunisation. (caregiver Duke of York Rural - rural)

##### Stage 2: intent

Some caregivers cited having a healthy child as motivation for immunising their children.

Well, I don’t want my baby to get sick. That’s why I am consistent with my baby’s vaccinations […] it is very important to me and for my baby. (caregiver Duke of York Rural - rural)

Most healthcare professionals reported that inadequate human resources coupled with frequent vaccine and vaccine equipment stockouts impacts their ability to perform their job of vaccinating children and impacts motivation. This sometimes means there are not enough staff to provide community outreach services and maintaining health centre service provision is prioritised. However occasionally, static clinics are also cancelled when *“there is no staff and when (they) don’t have the vaccines […]”.*

##### Stage 3: preparation, cost and effort

Caregivers discussed the importance of family support and community-based services to increase and enable childhood vaccination uptake. One urban-based caregiver cited both family and spousal support as key to being able to vaccinate her child stating that *“help comes from my family and husband, that’s why I find it easy to bring my child to the hospital for vaccination and other health-related issues”.*

Healthcare professionals reported that increased operational funds during immunisation campaigns leads to better coverage and enables a door-to-door approach. They also reported that liaising with community leaders ahead of mobile and outreach patrols led to more community members being available when they arrived in the community. Some healthcare professionals believe *“it is best we go and talk to our leaders ourselves, ward members and ward recorders. That we sit together, talk with each other to discuss and agree on something so it’s easy for us to go in look for those children and easily administer them their vaccines. Especially for those that live in the remote locations not the ones nearby”.*

Access barriers were the most common barriers experienced by caregivers in this stage (table 2). Many caregivers felt that geographical and financial challenges are key barriers to accessing routine immunisation services for their child(ren).

There is also a financial issue - bus fare. When I feel like I cannot afford this, I will just stay back with my child. I need money to go the clinic, and the hospital is far away. I need money for the bus fare and money for the clinic. I personally find it hard. (caregiver Toma-Vunadidir - rural)

Female caregivers face additional access challenges including difficulty finding childcare and not feeling safe when travelling to health facilities. One rural caregiver in Duke of York Rural reported that *“[…] when we have to walk to the health centre, sometimes it is not safe”.* Safety is also a key issue for healthcare professionals. In some areas law-and-order issues create additional obstacles to healthcare professionals vaccinating children in the community. One urban-based healthcare professional explained one instance when there was a fight, *“the police had to be there. […] Not safe. So they had to stay. So children missed out”.*

Healthcare professionals also highlighted that some children are missed due to population movement from intermarriages and plantation work.

They miss out on getting their immunisations when they move out of here and go out to their plantations. By the time they come back for us to serve them some are already overdue by 6 months or so. (healthcare professional Inland Baining - rural)

One rural-based healthcare professional reported that having a vehicle is a key enabler for outreach, making it *“very easy to go further in and reach the remote children for the routine immunisations”.* However, healthcare professionals described how their ability to provide routine immunisation services is significantly impacted by the health facility’s finances. Not having facility-owned vehicles, boats or funds to purchase fuel was often reported as a reason they were unable to conduct mobile or outreach activities.

##### Stage 4: point of service

Healthcare professionals report that understanding why vaccination is important for child health is an important enabler and when not explained by healthcare professionals, can lead to mothers giving up on trying to vaccinate their children. This was supported by a caregiver in Duke of York Rural who stated that “*sometimes, we mothers bring our babies for routine immunisation only and no awareness to us; that’s why some mothers do give up. They don’t know the reason for the baby’s vaccines, and also most mothers are village women who don’t understand the purpose of routine immunisation”.*

Another key enabler for caregivers highlighted by both healthcare professionals and caregivers was the door-to-door approach. This door-to-door approach enables them to “*[…] easily find those who haven’t received (a vaccine)”.* Most caregivers found that the door-to-door approach used during immunisation campaigns was the most effective way for healthcare professionals to find unvaccinated babies as it often captures children of caregivers who do not have financial means to travel to health facilities.

For caregivers who were able to overcome barriers in the first three stages, many encountered barriers at point of service. Vaccine stockouts were frequently reported with caregivers walking long distances to health facilities only to be *“[*…] *told that there is no vaccine in stock, and they are sent all the way back with no immunisation and that is why they don’t want to go”.* Some caregivers also highlighted that healthcare professionals often do not explain why children should be vaccinated, with one rural-based caregiver stating that “*they only administer it and tell us to come back later on with no explanation (of) its purpose”.* This emphasises the importance of supporting vaccine knowledge and awareness.

Not informing the community prior to outreach services often means that children in the community are not available to receive routine vaccines. Caregivers may be busy working and *“[…] tend to miss out last minute when (they) find out”.* When providing routine immunisation services, some healthcare professionals felt that there needed to be more staff trained to provide maternal and child health services. One healthcare professional explained how at their facility, non-maternal and child healthcare professionals lack confidence to carry out immunisation activities when maternal and child health professionals are absent.

For instance, if the maternal child health staff do not come to work or our duty roster is affected. […] if myself or the other nurse are off on the maternal child health clinic day and the other staff are there but are not confident enough to handle the babies, that’s when we cancel the immunisations. (healthcare professional Inland Baining - rural)

##### Stage 5: experience of care

Caregivers reported satisfaction with village-based immunisation services as an enabler, with one rural-based caregiver stating that *“it is easy for us because they came to the village and gave babies their vaccines and it is good for us. We want them to come; they need to come to the village every time”.*

Some caregivers feel judged by healthcare professionals when they take their child(ren) to get vaccinated. Caregivers cited that *“they fear nurses will get on them because they don’t have a clinic book or their records in the child’s book are not consistent […]”.* Two caregivers felt that vaccine stockouts impact caregiver trust in healthcare professionals.

One big reason is that they do not trust the doctors or nurses or health workers because most times when they go for their routine immunisation, they are told the vaccine is out of stock. (caregiver Duke of York Rural - rural)

Knowledge of vaccines and vaccine preventable diseases reportedly impacts caregivers’ experience of care when seeking vaccination services for their child(ren). Healthcare professionals want refresher immunisation training to ensure that they have the necessary and most up-to-date skills to deliver routine immunisation services. One rural-based healthcare professional emphasised that *“if more (healthcare professionals) go for training, they will be able to explain to the mothers what the mothers need to know […]”.*

##### Stage 6: after service

Both healthcare professionals and caregivers described recording the next date of vaccination in the child health book as an enabling mechanism that helps caregivers know when to bring their child back. Most caregivers reported they look to this to know when to return for their child’s next vaccination. When asked about why some children miss vaccines, a rural-based caregiver stated that *“it could be like that where they did not get proper information on the next date of appointment for immunisation dose, so they do not go to get it”.* Some healthcare professionals also highlighted that caregivers have limited understanding of vaccine side effects and how to manage these.

Sometimes, some of the mothers when they get the babies to the clinic, they get the first dose, and babies are very sick and fever, they blame the vaccine. Then the rest of the vaccines are not given. (Healthcare professional Kokopo Urban – urban)

### Strategies suggested by caregivers and healthcare professionals

Within stage one, participant responses focused on information, education and communication and mobilising village members to promote vaccination (table 4). Community leaders are key to raising awareness among the community. One urban-based health professional stated that “*(community leaders) need to support by giving awareness. Leaders need to know the reasons why parents are not bringing their children to the clinic site, and they need to know the importance of being vaccinated”.* Training key village members on routine vaccine promotion means that they can become “*a mouthpiece, eyes and ears at the community”.*

**Table 4:** Strategies suggested by caregivers and healthcare professionals

Journey to health and immunisation framework stage	Focus area	Strategies for community and religious leaders	Strategies for healthcare professionals	Strategies for governing authorities
Stage 1: knowledge, awareness and beliefs	Information, education and communication	Use a variety of communication channels to inform, educate and communicate with caregivers (ie, posters, individual education sessions with caregivers, community-led education sessions) Disseminate routine immunisation information at community and religious gatherings using these information, education and communication resources	Use a variety of communication channels to inform, educate and communicate with caregivers at health facilities and in the community (ie, posters, individual and group education sessions with caregivers) Provide information on potential side effects and the management of these	Develop tailored information, education and communication materials using findings from this study’s surveys and interviews. For example, flipcharts for healthcare professionals and community and religious leaders to educate caregivers and community members about routine immunisation
	Village members	Involve village members to help pass information from the community back to healthcare professionals Involve village members in promoting routine immunisation to help address language barriers^28^	Support village members to educate their community and use information from village members to target areas of under-immunisation	Provide financing required to run outreach services
Stage 3: preparation, cost and effort	Coordination between key stakeholders	Partner with healthcare professionals to bring services to the community Help inform healthcare professionals where missed children are likely to be located	Healthcare professionals support community and religious leaders in informing caregivers about routine immunisation service dates	Strengthen communication between the district health management team and health facilities regarding vaccine availability
Stage 4: point of service	Human resources	N/A	N/A	Increase staffing in facilities with human resource gaps
	Health infrastructure and vaccine supply	Work with the health authority to advocate for stronger health infrastructure	Work with the health authority to advocate for stronger health infrastructure	Open new aid posts and re-open non-functioning aid posts in remote areas to improve access for remote communities Strengthen vaccine supply chain
	Routine immunisation training	N/A	Provide routine immunisation training to community and religious leaders, including Ward Development Committees, so they are empowered to disseminate information to communities. (eg, Vaccine Champions education and communication programme recently rolled out in Fiji)^28^	Provide training across all healthcare provider levels. Training to include communication skills and refresher of Essential Programme on Immunisation including adverse events following immunisation (eg, Vaccine Champions education and communication programme recently rolled out in Fiji)^28^
	Village-based services	Advise community when outreach sessions will occur and support to access these services	Strengthen planning/delivery of routine immunisation outreach services at the village level to reduce access barriers such as transport costs and travel time	Strengthen the Village Health Assistant programme

Within stage three, participant responses focused on coordination between stakeholders (table 4).

I think health workers should inform [community and church leaders] so they can [announce] to us that [we] will be expecting you. (caregiver Bitapaka -rural)

Participant responses focused on human resources, health infrastructure and vaccine supply, routine immunisation training and village-based services within stage four (table 4).

To improve vaccination coverage here we need permanent staff based on [maternal child health]. (healthcare professional Toma-Vunadidir - rural)

I think health workers should come to our place and administer vaccines because sometimes I am busy, or my budget is a bit tight. (caregiver Bitapaka - rural)

## Discussion

This qualitative study of caregivers and healthcare professionals in ENBP identified a strong demand for comprehensive information about the purpose and impact of vaccination, bidirectional coordination and communication between healthcare professionals and community and religious leaders about routine immunisation, and community-based services to improve accessibility. It also highlighted the challenges faced due to health system infrastructure and human resource gaps, and in ensuring recruitment approaches are able to facilitate the inclusion of zero-dose children and their families. The findings highlight that communities would welcome healthcare professionals providing comprehensive vaccine information and services at the community level and that community members would like to become more involved in advocating for routine childhood immunisation. Understanding the context-specific enablers and barriers described in this study is an important step to inform effective action to bridge the vaccine inequity gap that leaves children unnecessarily susceptible to vaccine preventable diseases.

Caregivers reported a desire to understand the purpose of vaccines and a preference to have this more thoroughly explained by healthcare professionals. Caregiver and healthcare professional participants suggested communication interventions to improve vaccine knowledge, including locally tailored information, education and communication materials, and training on their effective delivery, can help address individual perceptions of vaccine risks and benefits, as well as perceptions of the vaccine provider.^28^,^31^ In Fiji, vaccine education and communication training for healthcare workers and community and religious leaders improved participants’ knowledge of effective communication skills and increased community members’ intention to vaccinate.^28^ Although ENBP has a high literacy rate of 86.6%, future communication interventions need to ensure the use of accessible and understandable resources that can be delivered in verbal and written form, to meaningfully address the health literacy gaps identified by caregivers in this study.^32^,^34^

Coordination and collaboration between healthcare professionals, community and religious leaders and the broader community is a strategy highlighted by study participants to increase community-level vaccine knowledge and uptake (figure 1). This approach was shown to improve vaccine knowledge and acceptance among a community with low vaccine uptake in a rural district in India.^31^ The involvement of religious leaders increased caregiver willingness to travel to vaccinate their children.^31^ A study conducted in rural and urban areas of Nigeria found that involving community leaders in building demand for immunisation improves acceptance and uptake.^35^ Our findings, along with these studies, demonstrate the importance of coordination and collaboration between healthcare professionals, community and religious leaders and the community to improve vaccine knowledge and uptake.

Access barriers identified by caregivers included financial and travel challenges at the individual level, and limited availability of healthcare professionals at the health system level (figure 1). These are often described in studies examining access to routine childhood vaccines across PNG and in other settings.^936^,^38^ Findings from this study, along with others from similar settings, support the suggested strategy by caregivers and healthcare professional participants of strengthening village-based vaccination services.^39^,^43^ A review of community-based interventions to improve coverage of childhood immunisation in low- and middle-income countries found that immunisation outreach alone, outreach in combination with non-monetary incentives and outreach in combination with immunisation education all likely improve vaccination schedule completion among children aged under five in urban and rural areas.^39^ Another study reported that using adjusted immunisation programming with outreach sessions conducted on the weekend increased vaccination coverage in urban areas of Uganda.^43^ These data highlight a need to refocus efforts and resources to implement innovative community-based strategies with a flexible approach to timing and location of service delivery.

Both caregivers and healthcare professionals in this study identified that broader governance and health system factors have a major impact on immunisation availability and access (figure 1). This finding is similar across many settings and supports the need for increased and sustainable financial resourcing for immunisation staff, health infrastructure and vaccine supply to enable greater vaccine coverage, and increased advocacy from all levels.^11 36 37 44 45^ Minimising vaccine stock-outs remains an important area of focus given decreased overall vaccine coverage leaves the population susceptible to vaccine preventable diseases and reduces community trust in immunisation programmes.^45^ To support advocacy efforts, community leaders, healthcare professionals and health authority leaders need to be resourced with evidence that can be translated into policy and used for advocacy. To do this, coupling research with implementation activities can enable further insights into the effectiveness of interventions aimed at increasing vaccination coverage.

### Strengths and limitations

Although this study was undertaken in areas with variable DTP1, DTP3 and MR1 coverage, almost all caregivers who participated in this research reported that their children had received DTP1, DTP3 and MR1 vaccines. The larger cross-sectional study this qualitative work was nested within found that 10% of children did not receive or had no record of receiving DTP1, and around 25% for MR1.^18^ Several factors may have influenced this divergence, including inaccuracies in the data describing the immunisation coverage in these areas and biases within the sampling frame.^46 47^ For example, caregiver survey participants who had not vaccinated their child may have chosen not to participate in an interview for fear of judgement from the researcher who was also a healthcare professional. For those who did participate, answers may be influenced by social desirability bias, with participants potentially reporting more socially desirable vaccination behaviours for caregivers or practices for healthcare professionals during interviews. Communities which tend to have lower immunisation coverage and may be harder to reach might have been less likely to be able to participate. Therefore, despite the myriad of enablers and barriers identified, these data may not capture the perspectives of caregivers of zero-dose and under-immunised children.

Participant answers may also be influenced by confirmation bias, with interviewers potentially probing and responding in a way that confirms their pre-existing beliefs. The qualitative research methods training included a discussion to reflect on beliefs and expectations that may influence the interview to try and reduce this presence of confirmation bias. Some prespecified areas were unable to be included due to security concerns. The impact of this missing data is uncertain. Financial and logistical constraints meant the research team was unable to travel to very remote locations which are more challenging to access, limiting our interpretation of the applicability of these findings to similar areas, although access is likely to remain a significant factor. These geographic accessibility and resourcing challenges impacted the number of participants recruited in each area, with more interviews than estimated occurring in some areas and fewer in others. However, data were collected from urban, rural, inland and small island communities which enables some applicability to similar settings. Due to the modality of interviewing, barriers were drawn out more than enablers through probing. Further exploring what enabled these caregivers to overcome barriers by ascertaining deeper insights into the factors and mechanisms that allowed them to navigate challenges to having their child immunised would be a valuable area to focus on in future studies. Although these results are specific to the ENBP context, it is likely that similar barriers and enablers to routine childhood immunisation are present in other areas of PNG, and this could be further explored.

## Conclusion

Findings described in this study indicate that implementing context-specific strategies that increase caregiver and healthcare professional immunisation knowledge, improve bidirectional coordination and communication between healthcare professionals and community and religious leaders about routine childhood immunisation, and strengthening community-based services to improve accessibility would likely increase routine childhood immunisation coverage. Strategies to strengthen community-based services and advocacy efforts aimed at increasing resourcing for immunisation staff, health infrastructure and vaccine supply are identified priorities by caregivers and healthcare providers alike. As a first step, these findings have informed the implementation of interventions in ENBP including the development of routine childhood immunisation communication materials and community leader and healthcare professional immunisation training.

## Supplementary material

10.1136/bmjph-2025-003553 online supplemental file 1

10.1136/bmjph-2025-003553 online supplemental file 2

10.1136/bmjph-2025-003553 online supplemental file 3

10.1136/bmjph-2025-003553 online supplemental file 4

## Data Availability

Data are available upon reasonable request.
