# Supplementary material for: Caregiver and healthcare professional perspectives on drivers of routine immunisation uptake in East New Britain, Papua New Guinea: a qualitative study
Source: BMJ Public Health. 2026 Mar 18;4(1):e003553. doi: 10.1136/bmjph-2025-003553 (PMC13007157; doi:10.1136/bmjph-2025-003553)
Supplement: online supplemental file 1 [file bmjph-4-1-s001.docx]

**Supplementary materials: interview guides**

**Caregiver Semi-Structured In-Depth Interview Guide**

We will now move to the last part of this interview. This part is a bit different to what we have just completed. We were asking questions with simple prepared responses. Now we want more in-depth information on your views and opinions of routine childhood vaccinations. I will now begin recording this part of the interview.

Nau umi bai go long laspela pat bilong dispela ol askim. Dispela ap bai inarapela kain liklik long wanem umi imekim nau tasol. Mipela ibin wok long askim sampela askim wer igat ol sotpela ansa wer yu iken givim. Nau, mipela i laikim moa infomesen long ol tingting bilong yu long ol wok bilong givim banis sut long ol pikinini. Mi bai stat nau long rekodim dispela ap bilong askim.

1. Do you know if there are any families in your village who have not vaccinated their children? Bai yu kilia sapos igat sampela femili insait long ples bilong yu wer ol pikinini bilong ol ino kisim banis sut or beibi sut?

If YES: What do you think are the main reasons children are not vaccinated?

Sapos yes: Long tingting bilong yu, yu ting wanem ol samting i mekim na ol dispela pikinini i no kisim banis sut?

Probes:

Ol toktok bilong kirapim tingting:

1. Lack of knowledge (Ino kilia tumas)
2. Transport issues to get to health services (Hevi long sait bilong kisim kar igo long kisim halivim long hausik)
3. Myths about vaccinations (Ol giaman toktok or stori long sait bilong banis sut or beibi sut)
4. Don't believe they are necessary (Oli nor ting olsem dispela emi bikpela samting)
5. Do not trust the healthcare workers (Noken putimbilip long ol helt wokmanmeri)
6. Law and order safety concerns (pasin bagarap insait long komyuniti)
7. Can you tell me how you would prefer to access routine vaccines for your child(ren)?

Inap yu tokim mi hau bai yu laik long kisim ol banis sut or beibi sut bilong ol pikinini bilong yu?

Probes:

Ol toktok bilong kirapim tingting:

1. Outreach patrols? Ol klinik patrol?

Definition: Involves health workers going to remote or hard-to-reach parts of an area and staying there for more than one night to deliver immunisations. This is routinely done and different to an immunisation campaign.

Tok kilia: Dispela em i toktok long ol helt wok manmeri igo long ol longwe ples o ples we ihat long go long em insait long wanpela ples na stap long hap, moa long wanpela nait long givim ol beibi sut or banis sut. Dispela emi save kamap olgeta taim na emi no wankain olsem kempein bilong banis sut.

1. Mobile clinics? Mobail klinik?

Definition: Organized within the catchment area of the health facility at popular/public locations (e.g., Church, School, Marketplace) - delivered by teams within one day.

Tok kilia: Dispela emi save stap insait long ples we ol lain isave bung long em long ol pablik ples (kain olsem ol Sios, Skul, Maket Ples) - we ol tim isave givim klinik sevis insait long wanpela dei.

1. At the health facility? Long helt senta?

Definition: Delivered at health centres and hospitals level, either daily or on fixed days, monthly or quarterly basis.

Tok kilia: Dispela em helt sevis we isave stap long ol helt senta or haus sik, na dispela isave kamap long olgeta dei o long ol de we oli makim long em, long wanwan mun o long wanwan kwota.

1. Can you tell me what it's like getting to immunisation services in your village?

Inap yu tokim mi long hau yu save kisim banis sut or beibi sut sevis insait long liklik ples bilong yu?

Do you find it hard or easy to access these services?

Yu save painim hat o isi long kisim ol dispela sevis?

1. Can you tell me what would help you stay up to date with immunisation?

Inap yu tokim mi long wanem samting iken helpim yu na yu bai inap long kisim olgeta banis sut?

Probe:

Ol toktok bilong kirapim tingting:

1. What helps them not miss doses or appointments? [Note: This is to probe for practical issues.]

Wanem samting isave halivim ol long inoken aburusim ol sut, marasin o taim bilong klinik?

1. Can you tell me how you would prefer to access information on routine vaccines for your child?

Inap yu tokim mi hau  bai yu laik long kisim infomesen long ol banis sut bilong ol pikinini?

Probes:

Ol toktok bilong kirapim tingting:

1. Ensuring information about day, time, and location of the immunisation sessions is reaching communities Sekim na lukim olsem ol toksave bilong dei, taim, na ples bilong banis sut iwok long go aut long ol komuniti.
2. Promotion of importance of vaccines in communities by community and religious leaders i.e., building vaccine confidence through community engagement and using vaccine champions Promosen bilong ol impoten banis sut insait long ol komuniti we ol manmeri na ol lida bilong komuniti na lotu iwok long mekim, kain olsem, wok bilong kamapim wanbel long banis sut taim ol ibung wantaim ol komuniti na yusim ol bikpla pes manmeri bilong banis sut.
3. What other support do you think would help families access immunisation services? Wanem ol narapela sapot yu ting bai iken halivim ol femili long kisim ol banis sut?
4. An immunisation campaign is when vaccinators have a one-off session to reach large groups of children with vaccines. For example, the polio vaccine campaign.

Banis sut or beibi sut kempein isave kamap taim ol lain bilong givim banis sut ibungim olgeta pikinini long wanpela bikpela grup na givim ol banis sut long ol. Wanpela tokbokis long sait bilong polio banis sut.

Can you tell me if a child in your care has benefited from an immunisation campaign?

Inap yu tokim mi hau wanpela pikinini we yu save lukautim ibin kisim sut o marasin long wanpela banis sut kempein?

If yes, can you tell me what you think works well during immunisation campaigns? What do you think does not work well during immunisation campaigns?

Sapos yes, inap yu tokim mi long wanem samting yu ting olsem emi gutpela long ol banis sut kempein?

1. Can you tell me how you think community and/or religious leaders could help increase routine immunisation uptake among caregivers?

Inap yu tokim mi long wanem we yu ting ol komuniti lida/bilong lotu lida iken mekim long halivim long mekim ol lukaut mamapapa long karim ol pikinini igo na kisim banis sut o marasin?

1. Can you tell me how you think healthcare workers could help increase routine immunisation uptake among caregivers?

Inap yu tokim mi long wanem we yu ting ol helt wokmanmeri iken mekim long halivim ol lukaut mampapa long karim ol pikinini igo na kisim banis sut o marasin?

1. Do you have any other beliefs or concerns about immunisation that you would like to discuss?   Yu gat ol narapela bilip o wari long?
2. We would like to provide information back to the community on this project as we continue. Can you tell me if you know of any good ways to do this?

Mipela iaik long givim infomesen igo bek long komuniti long dispela projek taim mipela igo yet. Inap yu tokim mipela sapos yu save long sampela gutpela wei bilong mekim dispela?

Probes:

Ol toktok bilong kirapim tingting:

1. Community meetings (Ol komuniti bung or toktok)
2. Health facility poster (Ol posta long ol hausik)
3. Flyer available at the health facility (Ol toksave pepe long hausik)

We have reached the end of this interview now. Thank you very much for your time and speaking with me about routine childhood immunisation. We will be using the findings from this research to support the Provincial Health Authority to strengthen routine childhood immunisation and will ensure that the community is given information on this project as we continue this work.

Mipela ikamap arere pinis long dispela ol askim. Tenkyu tru long taim bilong yu long toktok wantaim mi long ol banis sut bilong ol pikinini. Mipela bai yusim ol dispela ol wok painimaut long sapotim Provincial Health Authority long strongim ol wok bilong banis sut bilong ol pikinini na lukim olsem komuniti ikisim ol infomesen bilong dispela wok painimaut taim mipela igo hetlong wok.

**Caregiver Semi-Structured In-Depth Interview Guide**

We will now move to the last part of this interview. This part is a bit different to the sections we have just completed. Before, we were asking questions with simple prepared responses. Now, we want more in-depth information on your views and opinions of routine childhood vaccinations. I will now begin recording this part of the interview.

1. Can you tell me where children who have missed vaccinations are located approximately? Probe:
2. Villages
3. Can you tell me what you think is the best way to identify children who may have missed routine immunisations?

Probe:

1. Community or religious leaders
2. Ward counsellors
3. Ward health committee chair
4. Immunisation campaign coordinators
5. Immunisation records or coverage data
6. Do you think immunisation services are easy for families to access and use? Why or why not?
7. Can you tell me what you think is the best way to deliver routine vaccines to-hard-to-reach children?

Probe:

1. Outreach patrols?

Definition: Involves health workers going to remoter or hard-to-reach parts of an area and staying there for more than one night to deliver immunisations.

1. Mobile clinics?

Definition: Organised within the catchment area of the health facility at popular/public locations (e.g. Church, School, Marketplace) and delivered by teams within one day.

1. At the health facility?

Definition: Delivered at health centres and hospitals level, either daily or on fixed days, monthly, or quarterly basis.

1. Ensuring information about day, time, and location of the immunisation sessions is reaching communities.
2. Promotion of importance of vaccines in communities by community and religious leaders.
3. If you had the chance, what would you do to improve routine immunisation services in your area?
4. Can you tell me what you think is the biggest barrier for children receiving routine immunisations?

Probe:

1. Parent concerns
2. Lack of support from family and friends
3. Awareness of vaccination clinics/sessions
4. Not checking the vaccination card
5. Practical issues such as cost of travel
6. Outreach services not being provided
7. Law and order safety concerns
8. How common are cancelled static immunisation sessions?
9. Never
10. Very rare
11. Rare
12. Occasional
13. Frequent
14. How common are cancelled outreach immunisation sessions?
15. Never
16. Very rare
17. Rare
18. Occasional
19. Frequent
20. Can you tell me what you think the District Health Management team could do to improve vaccination coverage?
21. Do you know if people move in and out of the community regularly?

Probe:

a) Seasonal/plantation workers

b) Inter-marriages

1. Can you tell me how healthcare providers help encourage caregivers to vaccinate their children?
2. Can you tell me how you think community and religious leaders could help increase routine immunisation uptake among caregivers living in their communities?
3. Have you been involved in an immunisation campaign/supplementary immunisation activity (SIA)?

Probe if yes: What do you think works well during a vaccination campaign/SIAs?

Probe if no: What do you think does not work well during a vaccination campaign/SIAs?

Definition: An immunisation campaign/SIA are campaigns which have been used to rapidly scale-up coverage of key immunisations. They are primarily used for the distribution of polio and measles vaccines.

1. Would additional training be helpful to improve routine immunisation service delivery?
2. Can you tell me how COVID-19 has impacted routine service delivery in your area?
3. Do you believe community attitudes towards routine immunisations vaccination have changed because of COVID-19?
4. Do mothers or caregivers have any other beliefs or concerns about immunisation that you would like to discuss?

We have reached the end of this interview now. Thank you very much for your time and speaking with me about routine childhood immunisation. We will be using the findings from this research to support the Provincial Health Authority to strengthen routine childhood immunisation, so we look forward to keeping in touch as this project continues.
