# Supplementary figures and images for: Caregiver and healthcare professional perspectives on drivers of routine immunisation uptake in East New Britain, Papua New Guinea: a qualitative study
Source: BMJ Public Health. 2026 Mar 18;4(1):e003553. doi: 10.1136/bmjph-2025-003553 (PMC13007157; doi:10.1136/bmjph-2025-003553)

# UNICEF Journey to Health and Immunisation Framework

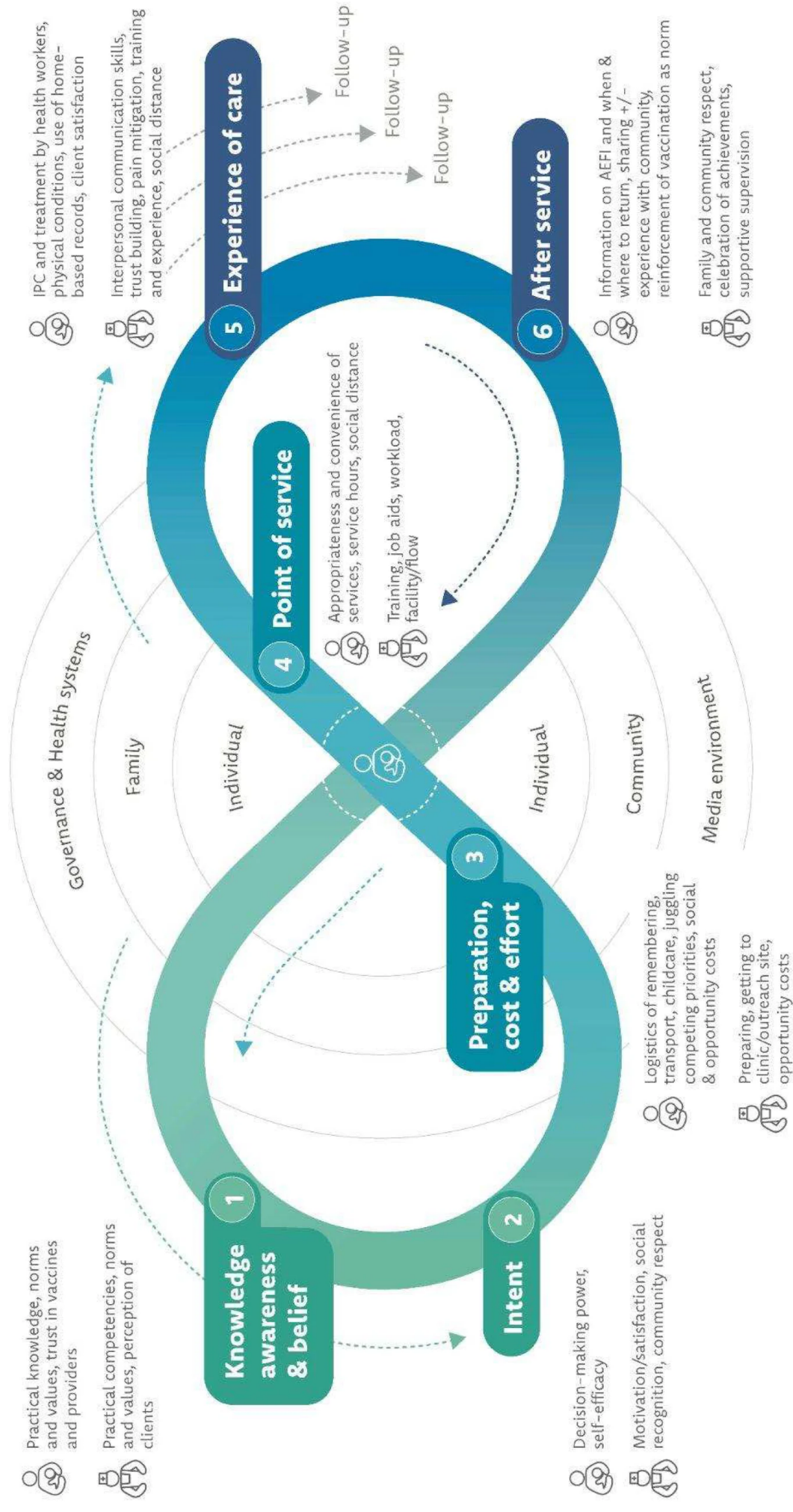

Supplement: online supplemental file 2 [file bmjph-4-1-s002.pdf]
