# Supplementary material for: Caregiver and healthcare professional perspectives on drivers of routine immunisation uptake in East New Britain, Papua New Guinea: a qualitative study
Source: BMJ Public Health. 2026 Mar 18;4(1):e003553. doi: 10.1136/bmjph-2025-003553 (PMC13007157; doi:10.1136/bmjph-2025-003553)
Supplement: online supplemental file 3 [file bmjph-4-1-s003.docx]

**Supplementary material:** Reflexivity statement

**Manuscript title:** Caregiver and healthcare professional perspectives on drivers of routine immunisation uptake in East New Britain, Papua New Guinea: a qualitative study

**1. Study conceptualisation**

***How does this study address local research and policy priorities?***

The idea for the Reaching Zero-Dose and Under-Immunised Children in East New Britain research program came from discussions with the ENB Provincial Health Authority (PHA) on its immunisation research priorities and built on previous research conducted in East New Britain Province (ENBP), which identified gaps in childhood immunisation coverage [1]. To develop this research program, planning and development work was conducted with primary care doctors, paediatricians and nurses working in the East New Britain health system as well as the PNG National Department of Health (NDoH), UNICEF Papua New Guinea (PNG) country office and the World Health Organization’s PNG country office. Funding was provided by the Australian Government through the Australian Non-Government Organisation Cooperation Program, which supports Burnet PNG country and partner driven programming.

***How were local researchers involved in the study design?***

As health systems strengthening was a core component of this research program, we used a co-creation and collaborative approach across organisations and expertise [2]. The investigator team included child health and immunisation experts from ENBP and Port Moresby, PNG, and Australia. This included the ENB PHA, the PNG Institute of Medical Research and the PNG NDoH. All members of the investigator team had input into the study design. This was managed through in-person workshops in ENBP with the PHA and online meetings which developed the protocol and then guided the study implementation plans.

**2. Research management**

***How has funding been used to support the local research team(s)?***

Study funding was used to provide quantitative and qualitative research training for the research officers who are based in PNG, both online and in person. It was also used to ensure that the PNG research team were able to travel to and collect data in study areas that experience conflict, safely. This included having security personnel travelling with the research team during field visits. Funding was also used to support the coordinating research officer (H.A.J.), a PNG national, to travel to the PNG Medical Symposium to present study findings.

**3. Data acquisition and analysis**

***How are research staff who conducted data collection acknowledged?***

All research staff who conducted the data collection and contributed to the interpretation of findings are authors.

***How have members of the research partnership been provided with access to study data?***

De-identified study data is available to all members of the research partnership and a report detailing the study data has been provided to all research partners in Papua New Guinea and Australia.

***How were data used to develop analytical skills within the partnership?***

Data were not used to directly develop analytical skills within the partnership, and we will prioritise this as an area to foster in ongoing and future work for this project. However, local research officers contributed to the interpretation and review of study findings, developing dissemination products and presenting study findings to different audiences (i.e. communities, PHA senior management, Project Advisory Panel, symposium attendees). Meetings were held online and face to face with authors where preliminary findings and frameworks were shared and feedback was provided to further develop the analysis.

**4. Data interpretation**

***How have research partners collaborated in interpreting study data?***

Preliminary study data were discussed in a series of consultative meetings and workshops with the research officer team, study investigator group, ENB PHA senior management and members of the Project Advisory Panel, comprising child health and immunisation experts from PNG and Australia. All these research partners provided critical oversight and input into the study implementation, approach and interpretation of findings. These changes were incorporated and re-circulated with the study team to ensure that the nuances had been included.

**5. Drafting and revising for intellectual content**

***How were research partners supported to develop writing skills?***

Research partners were not specifically supported to develop writing skills for this manuscript, and we will prioritise this as an area to foster in ongoing and future work for this project. However, the manuscript was developed and written as an iterative and collaborative process by all authors. All authors views and feedback were actively sought and have shaped the final paper. M.D. (Milena Dalton) worked closely with all authors during revisions to the manuscript to ensure all voices are represented.

***How will research products be shared to address local needs?***

Using the study findings, research officers went back to communities from which they collected data and disseminated study findings verbally at community consultative gatherings. We also held dissemination workshops in Kokopo, the capital city of ENBP with presentations detailing preliminary findings with the ENB PHA, healthcare professionals from the target health facilities and the Project Advisory Panel.

Research products including new information, education and communication materials informed by study findings have been developed and disseminated in the study areas. Training curricula for health workers has also been developed to address gaps in routine immunisation knowledge identified from this study, and training is currently being conducted with community leaders and healthcare workers in the study areas.

**6. Authorship**

***How is the leadership, contribution and ownership of this work by LMIC researchers recognised within the authorship?***

The authorship team includes a mix of early, mid and senior career researchers from both PNG and Australia. Of the 19 authors, eleven are Papua New Guinean and eight are Australian. All members of the research team that collected data and contributed to the interpretation of findings have contributed to this paper as co-authors. The lead investigator from the PNG Institute of Medical Research is second author on this paper.

***How have early career researchers across the partnership been included within the authorship team?***

Early career researchers (MD (Milena Dalton), HAJ, LD, DW, DM, BP and BS) contributed to this manuscript as authors. They were involved throughout the research and writing process. We acknowledge that they are based in both PNG and Australia.

***How has gender balance been addressed within the authorship?***

Seven authors identify as male (B.S., W.P., M.L., P.K., E.W., B.P., D.W.) and twelve authors identify as female (M.D. (Milena Dalton), M.D. (Margie Danchin), S.V., L.J.R., C.S.E.H., M.J.L.S., H.A.J., S.W., E.S., L.D., D.M. and P.M.).

**7. Training**

***How has the project contributed to training of LMIC researchers?***

The Reaching Zero-Dose and Under-Immunised Children in East New Britain research program provided quantitative and qualitative training opportunities for local Papua New Guinean researchers. This included training on conducting ethical research, community engagement prior to data collection, pilot testing research tools to ensure contextual relevance, electronic and paper data collection, data interpretation and sharing data collection findings back to participating communities.

**8. Infrastructure**

***How has the project contributed to improvements in local infrastructure?***

This project has not directly contributed to improvements in local infrastructure but has identified advocating for improved infrastructure as a key strategy for improving access to and uptake of routine immunisation.

**9. Governance**

***What safeguarding procedures were used to protect local study participants and researchers?***

All interviews were conducted in private locations and data de-identified. No identifying information is presented in the analysis. Community leaders and senior healthcare professionals in the study areas were consulted to select villages and permission to conduct research in these villages was granted by the ward councilor. The local research team was accompanied by security personnel when in the field collecting data.

**References**

1. Morgan, C. J., O. P. M. Saweri, N. Larme, E. Peach, P. Melepia, L. Au, M. J. L. Scoullar, M. S. Reza, L. M. Vallely, B. I. McPake*, et al.* "Strengthening routine immunization in papua new guinea: A cross-sectional provincial assessment of front-line services." *BMC Public Health* 20 (2020): 100.

2. Dori, A., R. Farquhar, T. Kelebi, E. Waipeli, Z. Kerry, S. Ruybal-Pesántez, D. Timbi, S. McEwen, L. Makita, M. Laman*, et al.* "Partnership-based approach to infectious disease research in papua new guinea." In *Transforming global health partnerships: Critical reflections and visions of equity at the research-practice interface*. A. Stewart Ibarra and A. D. LaBeaud. Cham: Springer Nature Switzerland, 2024, 133-46.
